# Chromosome-scale genome assembly of sweet cherry (*Prunus avium* L.) cv. Tieton obtained using long-read and Hi-C sequencing

**DOI:** 10.1038/s41438-020-00343-8

**Published:** 2020-08-01

**Authors:** Jiawei Wang, Weizhen Liu, Dongzi Zhu, Po Hong, Shizhong Zhang, Shijun Xiao, Yue Tan, Xin Chen, Li Xu, Xiaojuan Zong, Lisi Zhang, Hairong Wei, Xiaohui Yuan, Qingzhong Liu

**Affiliations:** 1 Shandong Key Laboratory of Fruit Biotechnology Breeding, Shandong Institute of Pomology, Taian, Shandong 271000 China; 2 School of Computer Science and Technology, Wuhan University of Technology, Wuhan, Hubei 430070 China; 3 State Key Laboratory of Crop Biology, Shandong Agricultural University, Taian, Shandong 271018 China; 4 Gooal Gene, Wuhan, Hubei 430070 China

**Keywords:** Genomics, Plant genetics

## Abstract

Sweet cherry (*Prunus avium*) is an economically significant fruit species in the genus *Prunus*. However, in contrast to other important fruit trees in this genus, only one draft genome assembly is available for sweet cherry, which was assembled using only Illumina short-read sequences. The incompleteness and low quality of the current sweet cherry draft genome limit its use in genetic and genomic studies. A high-quality chromosome-scale sweet cherry reference genome assembly is therefore needed. A total of 65.05 Gb of Oxford Nanopore long reads and 46.24 Gb of Illumina short reads were generated, representing ~190x and 136x coverage, respectively, of the sweet cherry genome. The final de novo assembly resulted in a phased haplotype assembly of 344.29 Mb with a contig N50 of 3.25 Mb. Hi-C scaffolding of the genome resulted in eight pseudochromosomes containing 99.59% of the bases in the assembled genome. Genome annotation revealed that more than half of the genome (59.40%) was composed of repetitive sequences, and 40,338 protein-coding genes were predicted, 75.40% of which were functionally annotated. With the chromosome-scale assembly, we revealed that gene duplication events contributed to the expansion of gene families for salicylic acid/jasmonic acid carboxyl methyltransferase and ankyrin repeat-containing proteins in the genome of sweet cherry. Four auxin-responsive genes (two GH3s and two SAURs) were induced in the late stage of fruit development, indicating that auxin is crucial for the sweet cherry ripening process. In addition, 772 resistance genes were identified and functionally predicted in the sweet cherry genome. The high-quality genome assembly of sweet cherry obtained in this study will provide valuable genomic resources for sweet cherry improvement and molecular breeding.

## Introduction

Sweet cherry (*Prunus avium*), originating from the Caspian and Black Sea regions, is an economically significant fruit species worldwide. Annual global sweet cherry production is ~2.2 million tons^1^. Sweet cherry was first introduced to China in the 1870s, and its widespread cultivation began in the 1990s. In the last year, the total planting area of sweet cherry in China reached 233,300 ha, with production of ~1.2 million tons^2^. In addition, there have been dedicated breeding efforts for this crop. The traditional breeding cycle of a sweet cherry cultivar is more than 15 years long, and marker-assisted selection and genomic selection are current candidate strategies for speeding up the breeding cycle^3^. Although sweet cherry has a simple, compact genome (2*n* = 2*x* = 16), only one draft genome assembly has been reported to date^4^. Owing to the use of short-read sequencing technology, the draft genome assembly exhibited a small scaffold N50 of 219.6 Kb and a low genome coverage of 77.8%.

Within the *Prunus* genus, the Chinese plum (Mei, *Prunus mume*) genome was first sequenced in 2012^5^. Since then, several other important fruit species have been sequenced, such as peach (*Prunus persica*)^6,7^, sweet cherry (*Prunus avium*)^4^, almond (*Prunus dulcis*)^8^, flowering cherry (*Prunus yedoensis*)^9^, a flowering cherry interspecific hybrid (‘Somei-Yoshino’, *Cerasus* × *yedoensis*)^10^, apricot (*Prunus armeniaca*)^11^ and European plum (*Prunus domestica*)^12^. All the sequenced genomes exhibit high coverage, ranging from 84.6% in Chinese plum^5^ to 126% in flowering cherry (*P. yedoensis*)^9^, with the exception of the genome assembly of sweet cherry cv. Satonishiki (77.8%)^4^. In other assemblies, including those of peach, almond, apricot, and Chinese plum, chromosome-level scaffolds with fewer gaps than in the assembly of sweet cherry cv. Satonishiki have also been constructed.

In this study, we aimed to assemble a high-quality chromosome-scale reference genome for sweet cherry using Oxford Nanopore technology (ONT) combined with short Illumina sequencing reads and Hi-C scaffolding. The high-quality genome assembly of sweet cherry will facilitate molecular breeding of sweet cherry and advance our understanding of the genetics and evolution of the *Prunus* genus.

## Results

### Sequencing and assembly of the sweet cherry cv. Tieton genome

The sweet cherry cultivar ‘Tieton’ (cv. Tieton) was used for whole-genome sequencing and chromosome-scale assembly. After filtering low-quality reads, a total of 65.05 Gb of Oxford Nanopore long reads and 46.24 Gb of Illumina short reads were obtained. The sequencing details are provided in Table S1.

Several approaches were applied to assemble the genome of sweet cherry cv. Tieton. The assembly statistics and Benchmarking Universal Single-Copy Orthologs (BUSCOs, v3.0.2)^13^ analysis results are shown in Table 1. The NECAT-medaka-NextPolish-Purge_Haplotigs assembly was chosen for further analysis because it showed the best quality. The genome assembly of sweet cherry cv. Tieton exhibited a total size of 344.29 Mb, consisting of 610 contigs with a contig N50 size of 3.25 Mb and a largest contig size of 13.6 Mb. The statistics for our sweet cherry cv. Tieton genome assembly are shown in Table 2.

**Table 1:** Statistics of different assemblies and BUSCO analysis results for the sweet cherry cv. Tieton genome

Assembly	Number of contigs	Contig N50 (kb)	Longest contig (kb)	Total contig length (Mb)	BUSCO analysis results				
					C	S	D	F	M
MaSuRCA (Flye)	2773	244.00	3148.23	322.30	98.1%	84.4%	13.7%	0.4%	1.5%
MaSuRCA (CABO)	1550	855.32	5752.65	336.99	98.0%	82.4%	15.6%	0.2%	1.8%
Canu	1863	715.83	10,366.47	409.75	67.2%	56.1%	11.1%	16.9%	15.9%
Canu-medaka-NextPolish	2013	737.80	10,558.92	417.34	98.0%	70.4%	27.6%	0.4%	1.6%
Canu-medaka-NextPolish- Purge_Haplotigs	462	1596.71	10,558.92	271.25	96.6%	91.6%	5.0%	1.1%,	2.3%
wtdbg2	9839	128.10	8345.69	467.82	88.0%	85.7%	2.3%	5.1%	6.9%
wtdbg2-medaka-NextPolish	7589	171.84	8398.19	409.70	98.0%	70.4%	27.6%	0.4%	1.6%
wtdbg2-medaka-NextPolish- Purge_Haplotigs	2959	402.20	8398.19	297.32	95.9%	92.3%	3.6%	0.7%	3.4%
NECAT	1417	2205.17	13,496.20	416.95	79.4%	69.7%	9.7%	11.2%	9.4%
NECAT-medaka-NextPolish	1390	2201.74	13,600.65	418.80	97.9%	83.9%	14.0%	0.3%	1.8%
NECAT-medaka-NextPolish- Purge_Haplotigs	610	3247.20	13,603.98	344.29	97.4%	91.2%	6.2%	0.5%	2.1%

For the BUSCO analysis results, complete BUSCOs (C), complete and single-copy BUSCOs (S), complete and duplicated BUSCOs (D), fragmented BUSCOs (F), and missing BUSCOs (M). MaSuRCA (Flye): MaSuRCA was used to generate the conscience super reads, and Flye was used to assemble the genome. MaSuRCA (CABO): MaSuRCA was used to generate the conscience super reads, and CABO was used to assemble the genome

**Table 2:** Statistics of the genome assembly for sweet cherry (*Prunus avium*) cv. Tieton

	Assembly	Pseudochromosomes
Assembled size (bp)	344,287,078	342,881,614
Number	610	8
NG50 (bp)	3,247,195	42,624,765
Maximum size (bp)	13,603,986	62,324,707
Minimum size (bp)	2627	30,632,009
Mean size (bp)	564,405.05	42,860,201.75
GC content (%)	38.44	38.43

**Fig. 1:**
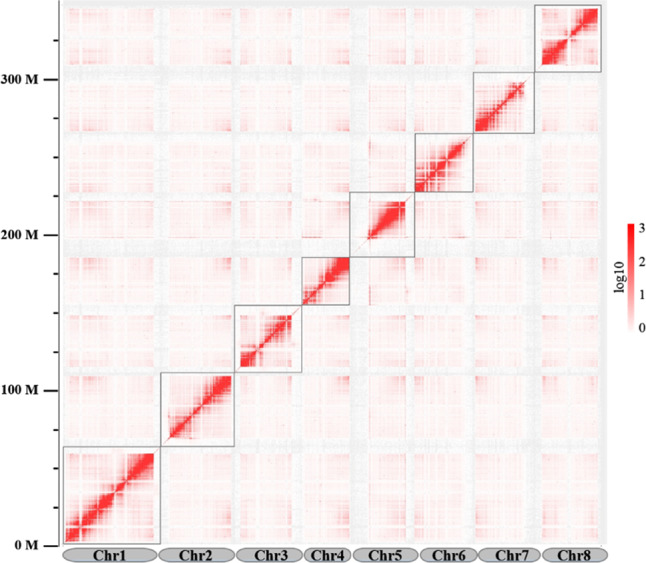
Hi-C interaction heatmap for the sweet cherry (*Prunus avium*) cv. Tieton genome. The map shows a high resolution of individual chromosomes that are scaffolded and assembled independently

Hi-C scaffolding generated a total of 134,298,049 read pairs (40.19 Gb) with a Q30 of 92.38%. After mapping the Hi-C reads against the assembly of sweet cherry cv. Tieton, 38.91 million valid interaction pairs, accounting for 82.12% of the unique mapped read pairs, were used for the Hi-C analysis. The statistics of the mapping details for the Hi-C data are shown in Table S2. ALLHiC v0.9.12^14^ was applied to construct the chromosomal-level scaffolds, and eight chromosomal-level scaffolds were generated, with lengths ranging from 30.63 to 62.32 Mb, accounting for 99.59% of the assembly. The statistics for the final pseudochromosomes of the sweet cherry cv. Tieton genome are shown in Table 2, and the Hi-C contact map is shown in Fig. 1. To the best of our knowledge, this is the first chromosomal-level genome assembly for sweet cherry.

### Genome assembly quality evaluation

The LTR assembly index (LAI), which was first introduced by Ou et al.^15^, is a newly developed reference-free genome metric that evaluates genome assembly continuity using long-terminal-repeat retrotransposons (LTR-RTs). The LAI is used to compare assembly quality between different species independent of genome size and total scaffold size, and its reliability has been proven in more than 40 plant genomes^15^. The LAI and contig N50 results for different genome assemblies within *Prunus* are shown in Table S3. Among the genome assemblies of sweet cherry cv. Tieton (this study), sweet cherry cv. Satonishiki^4^, peach v2.0^6^, flowering cherry (*P. yedoensis*)^9^, a flowering cherry interspecific hybrid (‘Somei-Yoshino’, *Cerasus* × *yedoensis*)^10^, Chinese plum^5^, almond^8^, apricot^11^, and European plum^12^, our sweet cherry cv. Tieton genome assembly shows the highest raw LAI (24.45) and LAI (19.68), nearly reaching the “gold standard” of genome assembly proposed by Ou et al.^15^. Among other *Prunus* species, only peach v2.0 (LAI = 18.79)^6^ and the newly assembled apricot genome (LAI = 16.29)^11^ exhibit similar scores, in agreement with their high assembly qualities. However, the previous genome assembly of sweet cherry cv. Satonishiki presented a raw LAI score of only 2.98 and failed to receive an LAI score due to its fragmented assembly.

The BUSCO analysis result, as shown in Table 1, implies the completeness of our sweet cherry cv. Tieton genome assembly. The NECAT-medaka-NextPolish-Purge_Haplotigs assembly identified 97.4% of the complete BUSCOs, corresponding to the greatest number of BUSCOs among the post-Purge_Haplotigs assemblies. Although the two MaSuRCA assemblies identified more complete BUSCOs, they also identified more duplicated BUSCOs.

As shown in Fig. S1, at kmers = 37, the sweet cherry cv. Tieton genome was estimated to be 340.05 Mb in size, with a heterozygosity of 0.409%, which is very close to the genome size of 338 Mb estimated by flow cytometry^16^. The slightly larger genome size of our assembly might be due to the heterozygosity of the sweet cherry genome. In addition, this result indicates the completeness of our cv. Tieton genome assembly.

### Annotation of the sweet cherry cv. Tieton genome

**Fig. 2:**
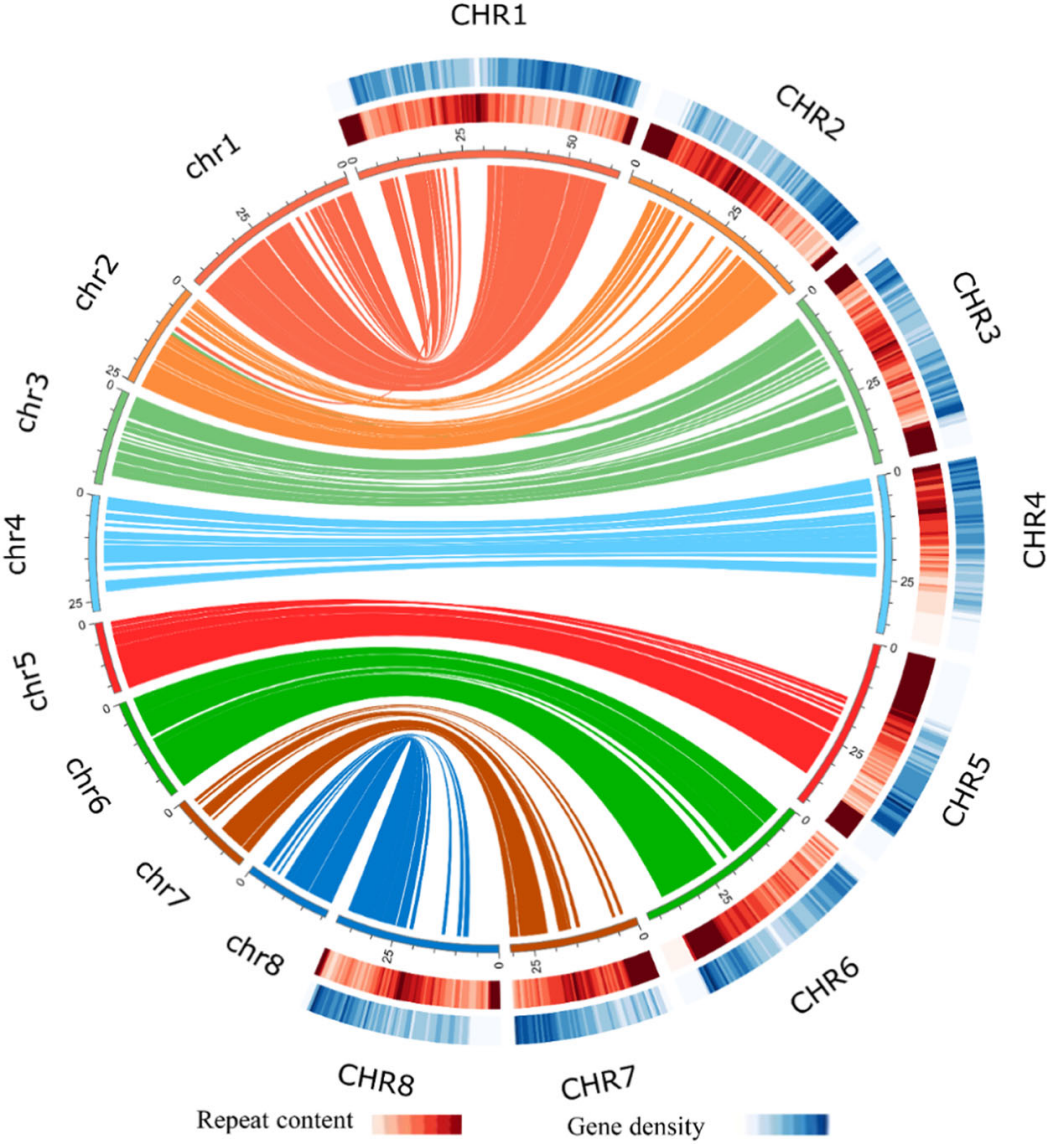
Characterization of sweet cherry cv. Tieton and its genome synteny with cv. Satonishiki. The innermost circle represents ideograms of eight pseudochromosomes of cv. Tieton (on the right side and labeled CHR1–CHR8) and the synteny relationship of gene blocks with the cv. Satonishiki genome (on the left side and labeled chr1–chr8). The middle half-circle represents the heatmap of the repeat content in the cv. Tieton genome. The outermost half-circle represents the heatmap of the gene density in the cv. Tieton genome

We used homology-based, de novo and RNA-seq methods for protein-coding gene prediction and functional annotation. A total of 38,275 gene models encoding 40,338 proteins were predicted in the sweet cherry cv. Tieton genome (Table 3 and Fig. 2). A total of 30,416 of 40,338 proteins (75.40%) were annotated by using the EggNOG v4.5.1^17^, Pfam v32.0^18^, UniProt v2019_10^19^, KEGG^20^, Gene Ontology^21^, COG^22^, BUSCO v2.0^13^, MEROPS v12.0^23^, Phobius v1.01^24^, SignalP v4.1^25^, and CAZyme v8.0^26^ databases or pipelines.

**Table 3:** Statistics of gene prediction and functional annotation for the sweet cherry (*Prunus avium*) cv. Tieton genome

Gene model statistics	Value	
Gene number	40,338	
Gene density (per 100 kb)	11.71	
Gene mean length (bp)	2758.67	
Exon number per gene	4.61	
Exon mean length (bp)	277.63	
Intron mean length (bp)	388.32	

Annotation database	Annotated number	Percentage (%)
EggNOG	28,711	71.18
InterPro	25,369	62.89
Pfam	21,349	52.93
KEGG	7536	18.68
COG	26,252	65.08
GO	18,198	45.11
UniProt	6217	15.41
BUSCO	1525	3.78
MEROPS	1007	2.50
Phobius	7699	19.09
SignalP	2727	6.76
CAZyme	1261	3.13
Sum	30,415	75.40

Compared with the former genome annotation of sweet cherry cv. Satonishiki^4^, our genome annotation of cv. Tieton predicted fewer gene models but with a longer total coding sequence (CDS) length. The CDS size distributions in the two genome annotations are shown in Fig. S2, and the statistics of the annotation profiles are shown in Table S4. According to Fig. S2, the genome annotation of sweet cherry cv. Satonishiki predicted more gene models with a length shorter than 650 bp, but our genome annotation of sweet cherry cv. Tieton predicted more gene models with a length longer than 650 bp. There were also more genes annotated by GO and KEGG pathways in our genome annotation of sweet cherry cv. Tieton (Table 3). These results suggest a better genome assembly and annotation of sweet cherry cv. Tieton than of cv. Satonishiki.

Repetitive sequence annotation showed a total content of 204.55 Mb, indicating that 59.40% of the sweet cherry cv. Tieton genome was repetitive (Table S5 and Fig. 2). Among these repetitive elements, LTR retrotransposons (19.71%) were the predominant component (8.50% Copia, followed by 7.59% Gypsy), whereas EnSpm (1.83%) was the most abundant class of DNA transposons. Our sweet cherry cv. Tieton genome exhibits a higher repetitive rate than the genome assembly of cv. Satonishiki (43.8%)^4^. The high repetitive rate in the sweet cherry genome may contribute to the poor genome assembly of sweet cherry cv. Satonishiki because short-read technology is limited in resolving highly repetitive sequences^27^.

The noncoding RNA prediction annotated 3825 noncoding RNAs, including 621 ribosomal RNAs, 1905 transfer RNAs, 1114 small nuclear RNAs, and 131 microRNAs, in the sweet cherry cv. Tieton genome (Table S6).

### Synteny analysis between the genome of sweet cherry cv. Tieton and the former draft genome of cv. Satonishiki

We performed a synteny analysis between the genomes of sweet cherry cv. Tieton and cv. Satonishiki, and the results are shown in Fig. 2. The genome of sweet cherry cv. Tieton shows good synteny with the genome of cv. Satonishiki. However, because of the fragmented genome assembly of cv. Satonishiki, many genes are not anchored to pseudomolecules, especially in high-repeat-content regions. This result also suggests the good quality of our genome assembly of sweet cherry cv. Tieton.

### Evolution of the sweet cherry genome

A series of evolutionary analyses were conducted by comparing the sweet cherry cv. Tieton genome with the genomes of other representative species in *Prunus*, including flowering cherry (*P. yedoensis*)^9^, peach v2.0^6^, almond^8^, apricot^11^, and Chinese plum^5^. Gene family clustering analysis assigned 172,683 proteins to 25,768 orthogroups, accounting for 92.6% (186,437) of the total proteins. The statistics for the orthology analysis results are shown in Table S7. There were 461 species-specific orthogroups in the sweet cherry genome, consisting of 2797 proteins. In addition, 816 orthogroups, accounting for 2850 proteins, were found to be shared between sweet cherry and flowering cherry. GO enrichment analysis of these genes revealed that catalytic and binding processes in the cellular component category and cellular and metabolic processes in the biological process category were the most enriched GO terms. The GO enrichment results are shown in Fig. S3.

The gene duplication event analysis revealed that sweet cherry and flowering cherry exhibited the largest numbers of gene duplication events, which were 10,618 and 9659, respectively (Fig. S4). However, 1329 gene duplication events were identified in peach, 4611 in apricot, and 3139 in Chinese plum. Although only 18,169 genes were identified in the almond genome^8^, 2989 gene duplication events were detected.

**Fig. 3:**
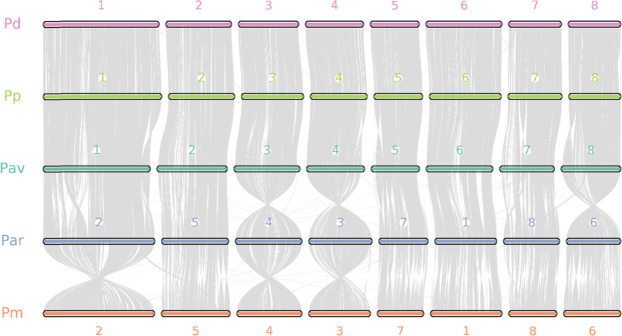
Chromosome-level collinearity patterns between sweet cherry cv. Tieton, peach, almond, apricot and Chinese plum. The numbers indicate the chromosome order generated from the original genome sequence. Each line represents one block. One block means that more than five paired genes were aligned in sequence. Pav: *Prunus avium* (sweet cherry), Pp: *Prunus persica* (peach), Pd: *Prunus dulcis* (almond), Par: *Prunus armeniaca* (apricot), Pm: *Prunus mume* (Chinese plum)

We also explored the genome synteny between the sweet cherry cv. Tieton and the other representative species. As shown in Fig. 3, our genome assembly of sweet cherry cv. Tieton exhibits a high level of genome synteny with all the other *Prunus* genomes, especially peach v2.0^6^. Only a few blocks were assigned to different chromosomes between our sweet cherry assembly and the peach assembly. Since peach is a genetically well-characterized model for research in *Prunus* species^28^ and high-level genome synteny is expected in this genus^29^, the highest genome synteny with peach v2.0^6^ suggests the good quality of our sweet cherry genome assembly.

The expansion and contraction of the gene families in sweet cherry cv. Tieton were analyzed by comparing the gene families with those in the other representative species, and the results are shown in Fig. S4. Compared with those in the other *Prunus* species, 1489 gene families in the sweet cherry cv. Tieton genome were expanded, and 1909 gene families were contracted. The sweet cherry and flowering cherry clade showed more gene family expansions and fewer gene family contractions than the other species in *Prunus*, with 1157 expanded gene families and 599 contracted gene families.

**Fig. 4:**
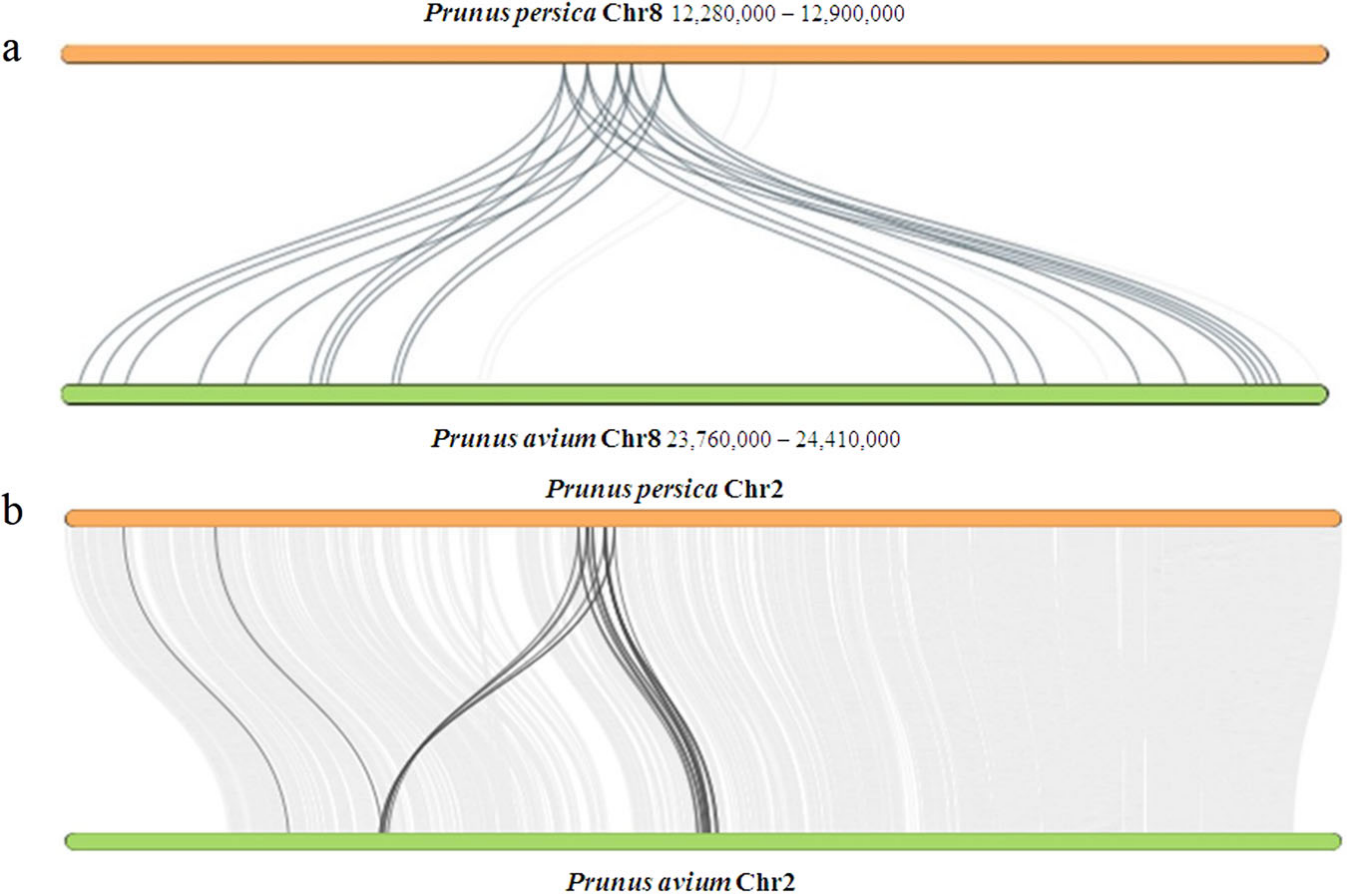
Collinearity patterns between the peach v2.0 (*Prunus persica*) and sweet cherry (*Prunus avium*) genomes. **a** Collinearity patterns of salicylic acid carboxyl methyltransferase (SAMT) on chromosome 8. The numbers indicate the chromosome position generated from the genome sequence. **b** Collinearity patterns of ankyrin repeat-containing (ANK) proteins on chromosome 2

Of the 1489 expanded gene families in the sweet cherry cv. Tieton genome, 108 were significantly expanded, consisting of 1125 genes (Table S8). These expanded genes exhibit diverse functions. The 121 genes (10.76%) were annotated in the KEGG (Kyoto Encyclopedia of Genes and Genomes) pathway database and were assigned to 37 pathways (Table S9). The five largest pathway categories were alpha-Linolenic acid metabolism (26 genes, ko00592), Ribosome (24 genes, ko03010), Spliceosome (21 genes, ko03040), Plant hormone signal transduction (11 genes, ko04075), and ABC transporters (7 genes, ko02010). In the alpha-linolenic acid metabolism pathways, 26 genes were identified as salicylate carboxymethyltransferase genes in the genome of sweet cherry cv. Tieton. These 26 genes were clustered with another 3 genes in sweet cherry into one gene family, which were identified as salicylic acid carboxyl methyltransferase (SAMT)/jasmonic acid carboxyl methyltransferase (JMT) genes. Only four genes in flowering cherry^9^, 11 genes in peach v2.0^6^, three genes in almond^8^, six genes in apricot^11^, and seven genes in Chinese plum^5^ were clustered in this gene family. Its expansion in the sweet cherry genome was mainly caused by two gene duplicate events occurring on chromosome 8. As shown in Fig. 4a, five of eight SAMTs in the peach genome were duplicated two times, corresponding to 20 of 24 SAMTs in the sweet cherry genome. SAMT converts salicylic acid to methyl salicylate, which acts as a long-distance mobile signal for systemic acquired resistance (SAR) in the uninfected part of the plant^30^. The expansion of SAMTs in sweet cherry suggests that salicylic acid may play important roles in the defense response against biotic and abiotic stresses. It is interesting that downstream nonexpressor of pathogenesis-related (NPR) genes in the salicylic acid signaling pathway were also identified in these significantly expanded gene families. In the plant hormone signal transduction pathways, 11 genes were annotated as ankyrin repeat-containing (ANK)-NPR-like proteins, which were clustered with 42 other ANKs in the genome of sweet cherry. ANKs constitute a large multigene family in higher plants and play important roles in protein–protein interactions in many developmental processes and abiotic and biotic stress resistance^31^. These 53 ANKs in the sweet cherry genome were clustered with 30 ANKs in flowering cherry^9^, 42 ANKs in peach v2.0^6^, 19 ANKs in almond^8^, 20 ANKs in apricot^11^, and 50 ANKs in Chinese plum^5^. The expansion of ANKs was probably caused by gene duplication on chromosome 2 because 35 ANKs in the sweet cherry genome and 19 ANKs in the peach v2.0 genome were detected on chromosome 2 (Fig. 4b).

### Fruit development-related genes in the sweet cherry genome

**Fig. 5:**
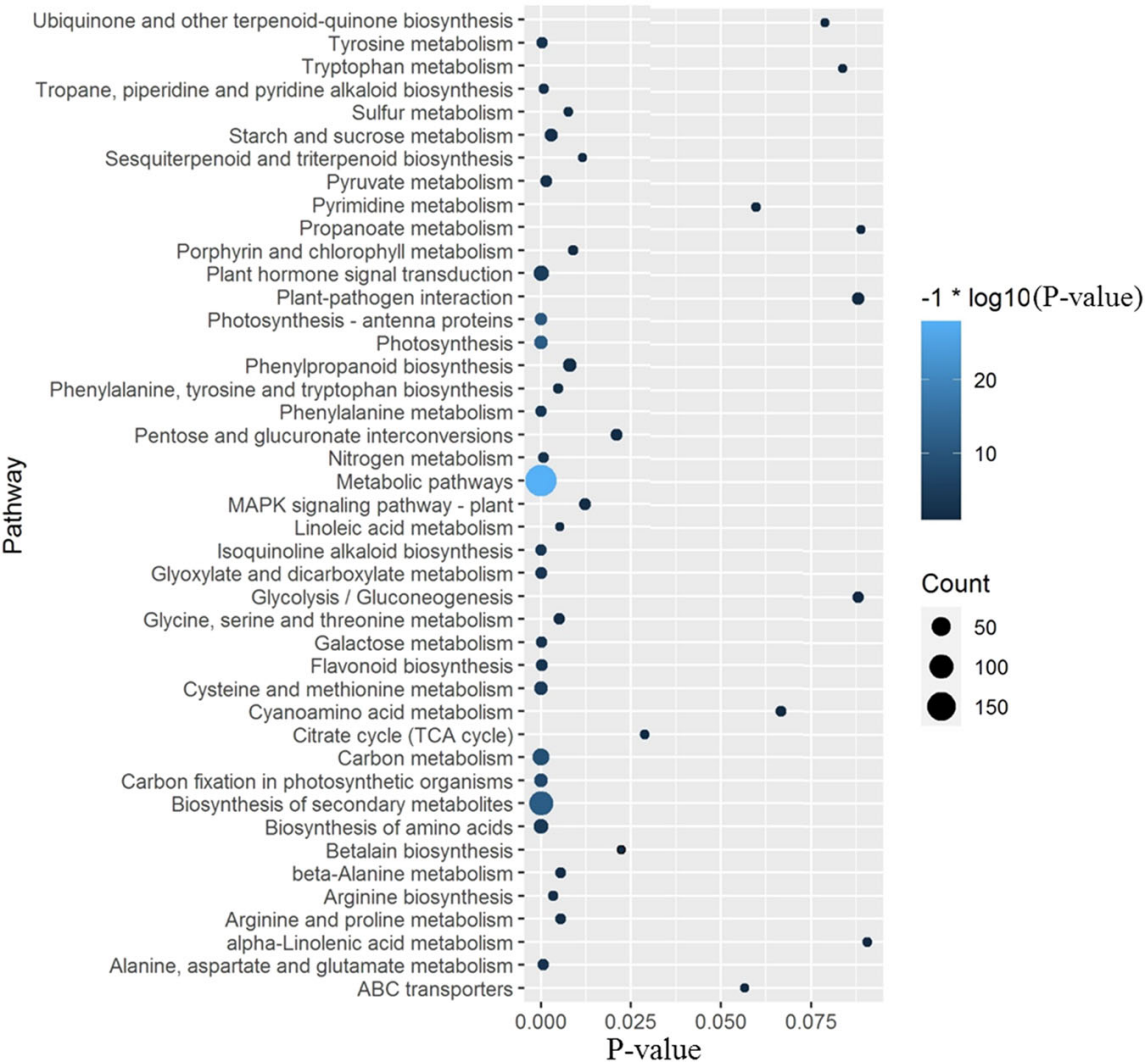
KEGG enrichment analysis of the differentially expressed genes during Tieton fruit development. The size of the dot represents the gene count. The color represents the *P*-value

Using our previously published transcript sequencing data for cv. Tieton fruit development^32^, we reanalyzed the sequencing data based on the assembled genome. A total of 1044 genes were identified as differentially expressed genes (DEGs, *P*-values < 0.01) during fruit development. The KEGG pathway enrichment analysis results of these DEGs are shown in Table S10, and the 43 largest categories are shown in Fig. 5. Among these pathways, metabolic pathways (pavi01100), photosynthesis (pavi00195), biosynthesis of secondary metabolites (pavi01110), photosynthesis-antenna proteins (pavi00196), carbon metabolism (pavi01200), carbon fixation in photosynthetic organisms (pavi00710), cysteine and methionine metabolism (pavi00270), plant hormone signal transduction (pavi04075), biosynthesis of amino acids (pavi01230), and Isoquinoline alkaloid biosynthesis (pavi00950) were the 10 largest categories (with the lowest *P*-values), indicating that these pathways play important roles in the fruit development of sweet cherry cv. Tieton.

**Fig. 6:**
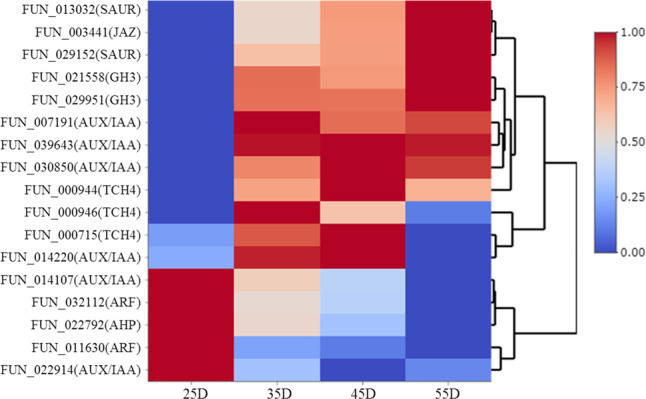
Expression patterns of plant hormone signal transduction pathway genes involved in sweet cherry fruit development. 25D: 25 days after flowering, 35D: 35 days after flowering, 45D: 45 days after flowering, 55D: 55 days after flowering

Among all the DEGs, 26 genes were involved in plant hormone signal transduction pathways. Twelve of them were involved in the auxin signaling pathway, and 9 of them were identified as abscisic acid (ABA)-responsive genes. In comparison with the well-known function of ABA-responsive genes in the fruit development of sweet cherry^33^, how auxin-responsive genes are involved in this process is still unclear. Of these auxin-responsive genes, six were identified as *AUX/IAA* (*auxin-responsive protein IAA*), two were identified as *GH3* (*indole-3-acetic acid-amido synthetase GH3*), two were identified as *SAUR* (*small auxin-up RNA*), and two were identified as *ARF* (auxin response factor). The expression patterns of these genes are shown in Fig. 6. It is known that the application of exogenous auxin can delay fruit development, and the endogenous auxin level decreases during the fruit ripening process in sweet cherry^34–36^. The gene expression patterns of the *AUX/IAA*s and *ARF*s were consistent with the endogenous auxin level during fruit development. However, the increased expression of *GH3*s and *SAUR*s in the late stage of fruit development reveals a complicated interaction between auxin and the sweet cherry ripening process. Further studies are needed to explore the function of auxin in fruit development in sweet cherry. Three members of *TCH4* (xyloglucan endotransglucosylase/hydrolase protein) involved in the brassinosteroid signal transduction pathway were identified as DEGs, which were mainly expressed in the middle stage of fruit development in sweet cherry. One jasmonate ZIM domain-containing protein (identified as *TIFY 9*) was increased during the fruit ripening process, which confirms that jasmonic acid is involved in this process in sweet cherry^37–39^. One *AHP* (histidine-containing phosphotransfer protein 1-like) was decreased during this process, consistent with the endogenous cytokinin level changes in sweet cherry fruit development^34^.

### Resistance-related genes in the sweet cherry genome

The sweet cherry cv. Tieton genome consisted of 772 resistance-related (*R*) genes with leucine-rich repeat (LRR) domains and/or nucleotide-binding sites (NBSs). These genes were classified into five groups: *coiled-coil-NBS-LRR* (*CNL*), *TIR-NBS-LRR* (*TNL*), NBS-LRR (*NL*), *LRR*, and *NBS*. *LRR* was the largest group, with 506 genes, whereas *CNL* included 148 genes, *TNL* included 31 genes, NL included 229 genes, and NBS included 44 genes. Compared to the peach genome, with 437 *R* genes, the sweet cherry genome had more *R* genes (772). The sweet cherry genome had more LRR domain-containing proteins, whereas the peach genome had more TNL-encoding genes (146 genes vs. 31 genes).

We also conducted chromosome-level genome synteny analysis of the *R* genes in the sweet cherry and peach genomes. As shown in Fig. S5, the majority of the *R* genes in the sweet cherry genome showed good synteny with those in the peach genome. In addition, these *R* genes were unevenly distributed along the eight chromosomes of sweet cherry. Their hotspots resided on chromosomes 1, 2, and 8, which are clearly shown in Fig. S6.

## Discussion

Using the combination of Oxford Nanopore technology, Illumina short reads and Hi-C scaffolding, we successfully assembled a high-quality reference genome for sweet cherry cv. Tieton and anchored 99.59% of the sequences to eight pseudochromosomes. According to the LAI-based method for assessing genome assembly quality, our sweet cherry cv. Tieton genome assembly shows better assembly quality than all the documented genomes within *Prunus*. This genome assembly can serve as a high-quality reference genome for sweet cherry to support studies of molecular breeding, genetics and evolution in sweet cherry and the *Prunus* genus.

Compared with the previous draft assembly of sweet cherry cv. Satonishiki, our genome assembly of cv. Tieton provides higher coverage and better continuity. First, the assembly size for sweet cherry cv. Tieton has been increased to 344.29 Mb, representing 101.2% of the estimated genome size. Second, our assembly of cv. Tieton exhibits a 15.47x longer contig N50 than cv. Satonishiki. We also annotated more genes than were included in the former annotation of cv. Satonishiki, suggesting the better continuity of our assembly.

The repetitive rate in the genome of sweet cherry cv. Tieton (59.40%) is higher than that in all the other sequenced species of *Prunus*, such as almond (34.6%)^8^, peach (37.14%)^7^, apricot (38.28%)^11^, Chinese plum (45.0%)^5^, and flowering cherry (*P. yedoensis*, 47.2%)^9^. There are also more gene duplication events in sweet cherry and flowering cherry (*P. yedoensis*) than in peach, almond, apricot, and Chinese plum. These results may explain why sweet cherry has a larger genome than other diploid *Prunus* species.

Two gene duplication events were identified in the genome of sweet cherry compared with peach v2.0, which were involved in the salicylic acid synthesis and salicylic acid signal transduction pathways. We also identified some auxin-responsive genes that were differentially expressed during the fruit development of sweet cherry. This finding suggests that auxin plays a complicated role in the fruit ripening process of sweet cherry. Our well-assembled reference genome of sweet cherry cv. Tieton will support studies on gene function characterization and benefit the research community in the future.

## Materials and methods

### DNA extraction and sequencing

Leaf samples from sweet cherry cv. Tieton grown in the experimental orchard of Shandong Institute of Pomology, Taian, China, were collected and frozen in liquid nitrogen. Genomic DNA (gDNA) was extracted, size selected and sequenced on an Oxford Nanopore PromethION system by BioMarker Technology Co., Ltd. (Beijing, China). Low-quality reads, reads with adapters and reads shorter than 2000 nt were filtered out. Before assembly, 2000 reads were randomly selected and subjected to BLAST searches against the NT database using BLAST v2.9.0+, and no obvious external contamination was found. For Illumina sequencing, a paired-end library with an insert size of 350 bp was constructed and sequenced by CapitalBio Technology Inc. (Beijing, China) in accordance with the manufacturer’s protocol using the Illumina HiSeq X Ten platform with a 150-nt layout. A total of 48.27 Gb of Illumina raw sequencing data was generated for the survey, correction and evaluation of the genome.

### Genome assembly

Several approaches were applied to assemble the sweet cherry cv. Tieton genome using MaSuRCA v3.3.4^40^, Canu v1.8^41^, wtdbg2 v2.5, and NECAT v0.01^42^. To facilitate reading, a complete set of parameters for each tool is listed in Supplementary Table S11. BUSCO v3.0.2^43^ was used to assess all the assemblies. The preliminary assemblies of Canu, wtdbg2, and NECAT were first polished with medaka (https://github.com/nanoporetech/medaka) using Nanopore long reads and then polished three times with NextPolish v1.0.5 using Illumina short reads. The three draft assemblies were subsequently processed with Purge Haplotigs v1.1.0^44^ to investigate the proper assignment of contigs.

### Hi-C analysis and pseudochromosome construction

Fresh young leaves collected from a sweet cherry cv. Tieton tree were fixed using formaldehyde at a concentration of 1%. The chromatin was cross-linked and digested using the restriction enzyme HindIII. The 5′ overhangs were labeled with a biotinylated tag and end repaired. After ligation, the DNA was extracted, sheared, and subjected to selection for fragments with lengths between 300 and 700 bp. The biotin-containing fragments were captured for library construction. Finally, the quality of the purified library was evaluated with a Qubit 3.0 fluorometer (Thermo Fisher Scientific, Inc.), quantitative PCR (qPCR), and an Agilent 2100 instrument. The qualified library was sequenced using the Illumina HiSeq X Ten platform with a 150-bp PE layout. To obtain the unique mapped read pairs, we first truncated the paired reads at the putative Hi-C junctions and then aligned them to the sweet cherry cv. Tieton genome assembly using the Arima-HiC Mapping Pipeline (https://github.com/ArimaGenomics/mapping_pipeline). We then used the partition, rescue, optimize, and build functions of the ALLHiC v0.9.12^14^ pipeline to construct the chromosomal-level scaffolds of the sweet cherry cv. Tieton genome. After ALLHiC scaffolding, pseudomolecules and Hi-C contact maps were built by using Juicer v1.6.2^45^ and the 3D de novo assembly pipeline v180114^46^. Juicebox Assembly Tools v1.9.8 were used to visualize and manually correct the large-scale inversions and translocations to obtain the final pseudochromosomes.

### Genome assembly quality evaluation

Following the approach of Ou et al.^15^, LTRharvest v1.6.1 and LTR_retriever v2.8 were first used to detect LTR-RTs in the genome assemblies, and then the LAIs of each genome were calculated using LTR_retriever. The genome size of sweet cherry cv. Tieton was estimated by using Jellyfish v2.2.10^47^ and GenomeScope v1.0^48^ based on a k-mer counting method.

### Repetitive sequence annotation

RepeatModeler v.2.0.1^49^ was applied as the de novo method to identify repetitive elements in the sweet cherry cv. Tieton genome. Then, the homology-based tool RepeatMasker v.4.0.9^50^ was applied against Dfam v3.1^51^ and Repbase library v20170127 using the Embryophyta settings.

### Noncoding RNA prediction

Infernal v1.1.2^52^ was used to identify noncoding RNAs (ncRNAs) in the sweet cherry cv. Tieton genome by comparison with the RFAM database v14.1^53^. tRNAscan-SE v2.0.5 was used to identify tRNAs. RNAmmer v1.1.2^54^ was used to search for rRNAs. The results of the three pipelines were combined, and the overlapping annotations were removed.

### Protein-coding gene prediction and functional annotation

Funannotate v1.7.2^55^, which employs GeneMark-ES v4.48_3.60_lic^56^, Augustus v3.3.2^57^, CodingQuarry v2.0^58^, SNAP v2006-07-28^59^, and GlimmerHMM v3.0.4^60^, was used to perform protein-coding gene prediction in the repeat-masked sweet cherry cv. Tieton genome. Briefly, we first trained the gene prediction model using our previously published transcript sequencing data for cv. Tieton fruit^32^. Then, protein-coding genes were predicted for the whole genome using the Funannotate prediction pipeline. All the predicted gene models, transcript alignments generated by PASA v2.4.1^61^, and protein sequences of the peach v2.0 annotation^6^ were input into EVidenceModeler v1.1.1 to obtain the final prediction of gene models for the sweet cherry cv. Tieton genome. The statistics of the prediction results and the weights used for EVidenceModeler are shown in Table S12. InterProScan5 v5.0^62^ was used to annotate the predicted proteins against the InterPro database v77.0^63^. EggNOG-mapper v1.0.3^17^ was used locally against emapperdb v4.5.1 for fast functional annotation with Gene Ontology^21^ and COG functional categories^22^. KAAS (KEGG Automatic Annotation Server)^64^ was used to annotate the predicted protein sequences according to KEGG Orthology. The results of the InterProScan, EggNOG-mapper, and KEGG Orthology analyses were combined and fitted to the Funannotate annotation pipeline. UTRs were added by the Funannotate update pipeline using the transcript data.

### Gene orthologs and gene family analysis

Gene orthologs and gene duplication events in the sweet cherry genome compared with the genomes of other *Prunus* plants were identified by OrthoFinder v2.3.11^65^. The GO enrichment analysis of these proteins was performed by using WEGO v2.0^66^. CAFÉ v4.21^67^ was employed to explore gene family size expansion and contraction following the provided protocol.

### RNAseq analysis and KEGG pathway enrichment of DEGs

RNAseq analysis was performed following the protocol of Thomas W. Battaglia (https://github.com/twbattaglia/RNAseq-workflow). TCC-GUI was used to normalize the expression data and detect the DEGs^68^. KOBAS 3.0 was used to perform the KEGG enrichment analysis^69^.

### Genome synteny analysis

A Python version of MCscan^70^ was employed to analyze the synteny between the sweet cherry cv. Tieton genome and the cv. Satonishiki genome or other genomes within *Prunus* following the approach of Haibao Tang. Circos v0.69-8^71^ was used to generate the synteny plot between sweet cherry cv. Tieton and cv. Satonishiki.

## Supplementary information

Supporting Information

Supporting Information 2

## Data Availability

Raw sequencing reads have been deposited in the NCBI SRA (Sequence Read Archive) database under BioSample accession number SAMN13640536. This whole-genome shotgun project has been deposited in GenBank under the accession JAAOZG000000000. The version described in this paper is version JAAOZG010000000.
