# Supplementary material for: Chromosome-scale genome assembly of sweet cherry (Prunus avium L.) cv. Tieton obtained using long-read and Hi-C sequencing
Source: Hortic Res. 2020 Aug 1;7:122. doi: 10.1038/s41438-020-00343-8 (PMC7395734; doi:10.1038/s41438-020-00343-8)
Supplement: Supplementary file 1 — Supporting Information [file 41438_2020_343_MOESM1_ESM.docx]

**Supplemental files**

**
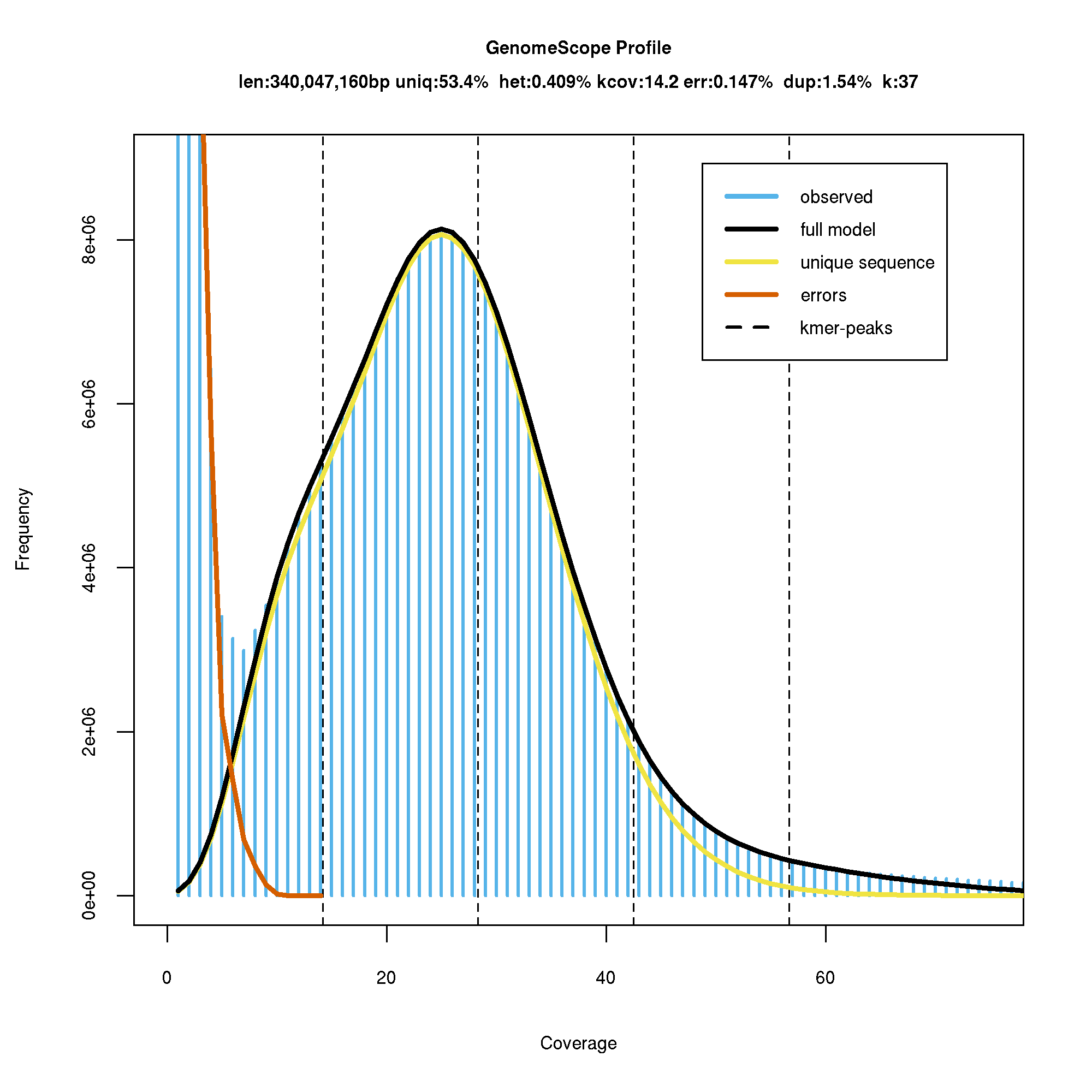
**

**Figure S1** K-mer (37-mer) plot of sweet cherry (*Prunus avium*) cv. Tieton for genome size estimation. A total of 16.79Gb Illumina clean reads representing 49x coverage of sweet cherry cv. Tieton genome was used for jellyfish analysis.


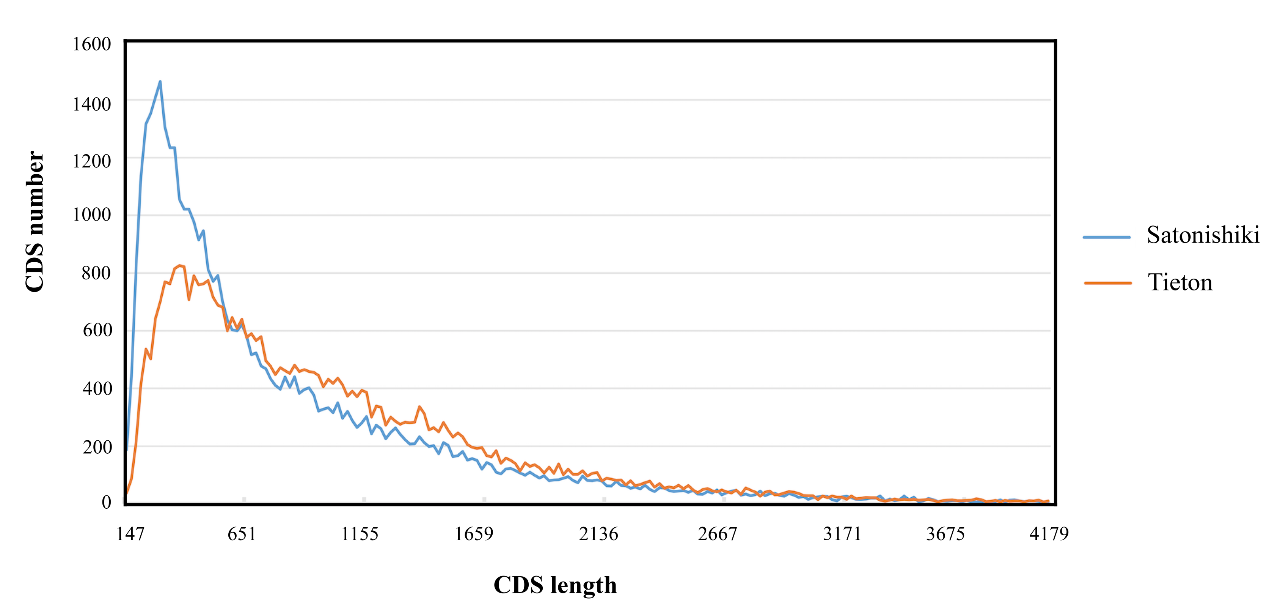


**Figure S2** CDS size distribution of genome annotation of sweet cherry cv. Satonishiki and cv. Tieton. X-axes shows the length (bp) of the CDS and Y-axes shows the count number.


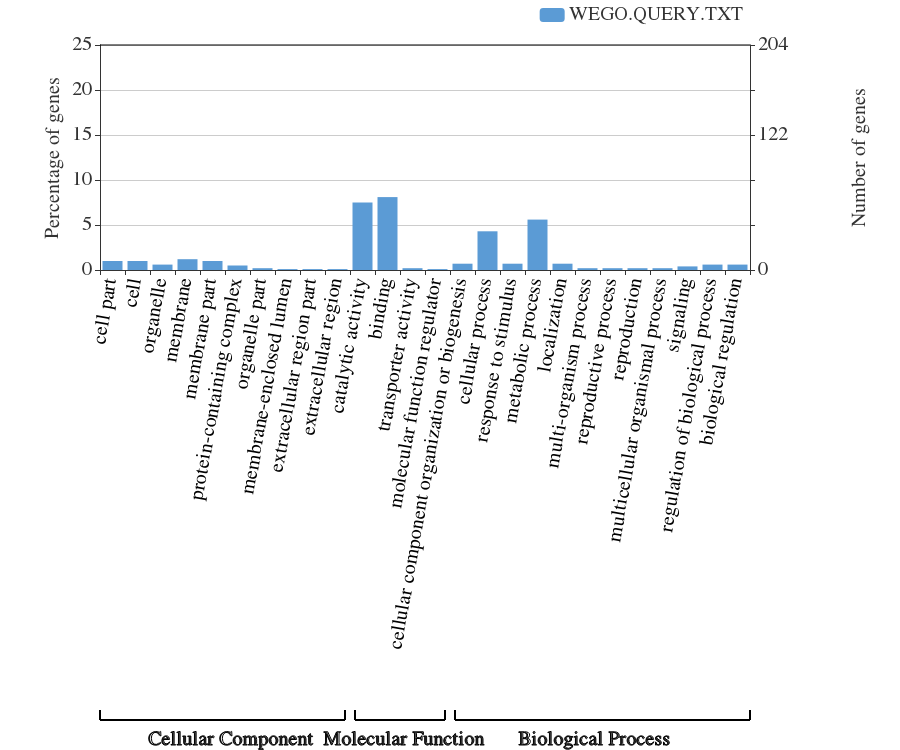


**Figure S3** GO enrichment analysis of 816 orthogroups shared by sweet cherry (*Prunus avium*) cv. Tieton and flowering cherry (*Prunus yedoensis*).


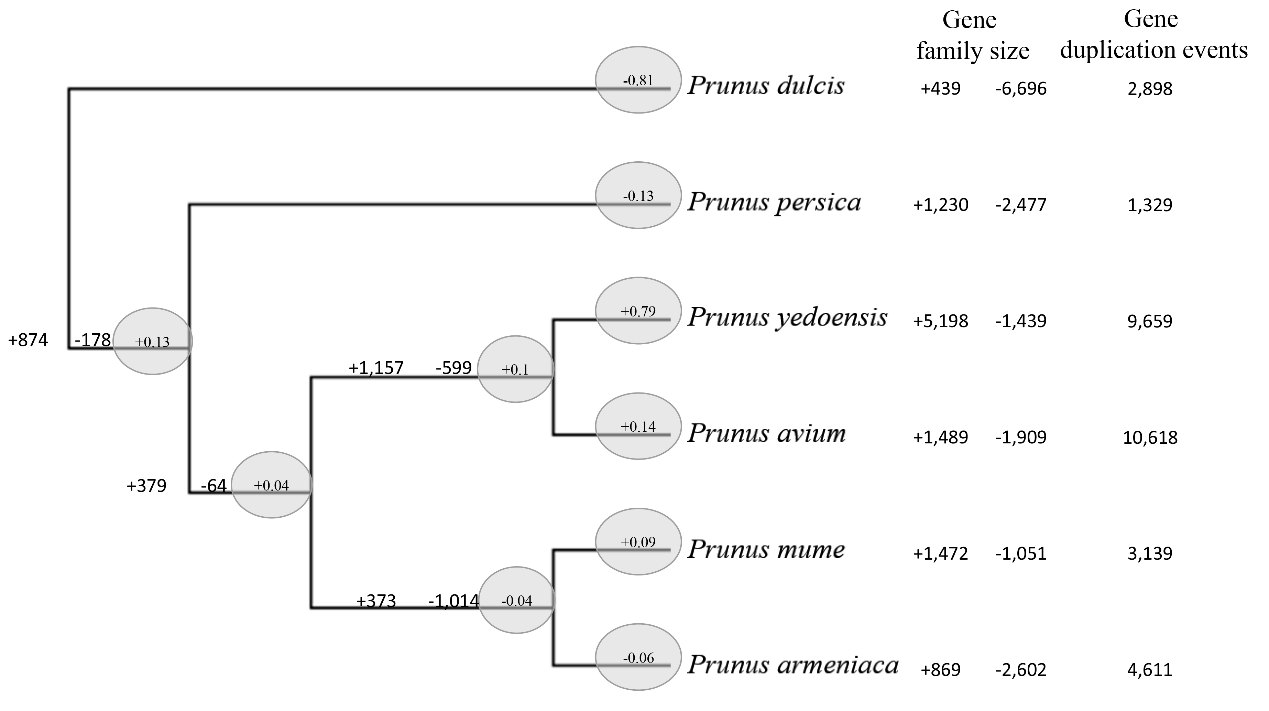


**Figure S4** Gene duplication events and gene family size analysis for sweet cherry (*Prunus avium*) cv. Tieton genome. Species tree was generated by STRIDE, as part of OrthoFinder. Species tree shows the estimated gene gain/loss rates (grey spot), the numbers of expanded/contracted families (+/-), and the numbers of gene duplication events.


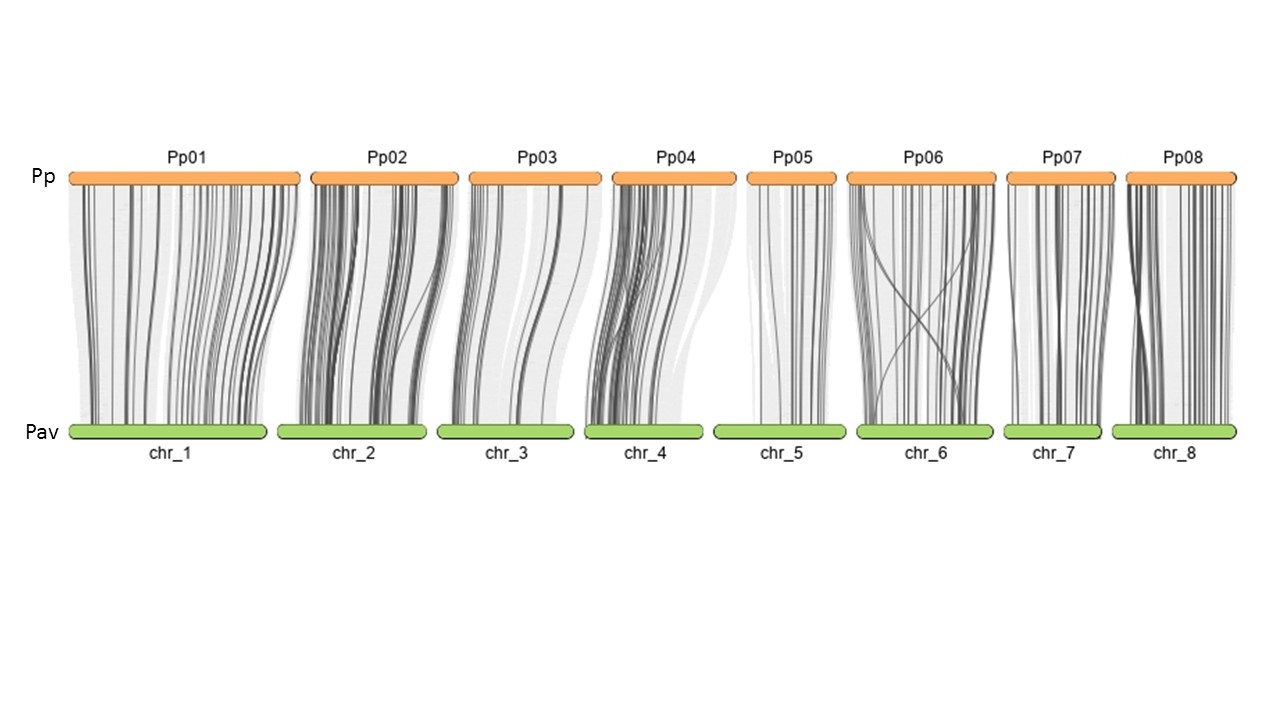


**Figure S5** Chromosome-level genome synteny of *R* genes between sweet cherry (*Prunus avium*) and peach (*Prunus persica*). Pav: *Prunus avium*, Pp: *Prunus persica*


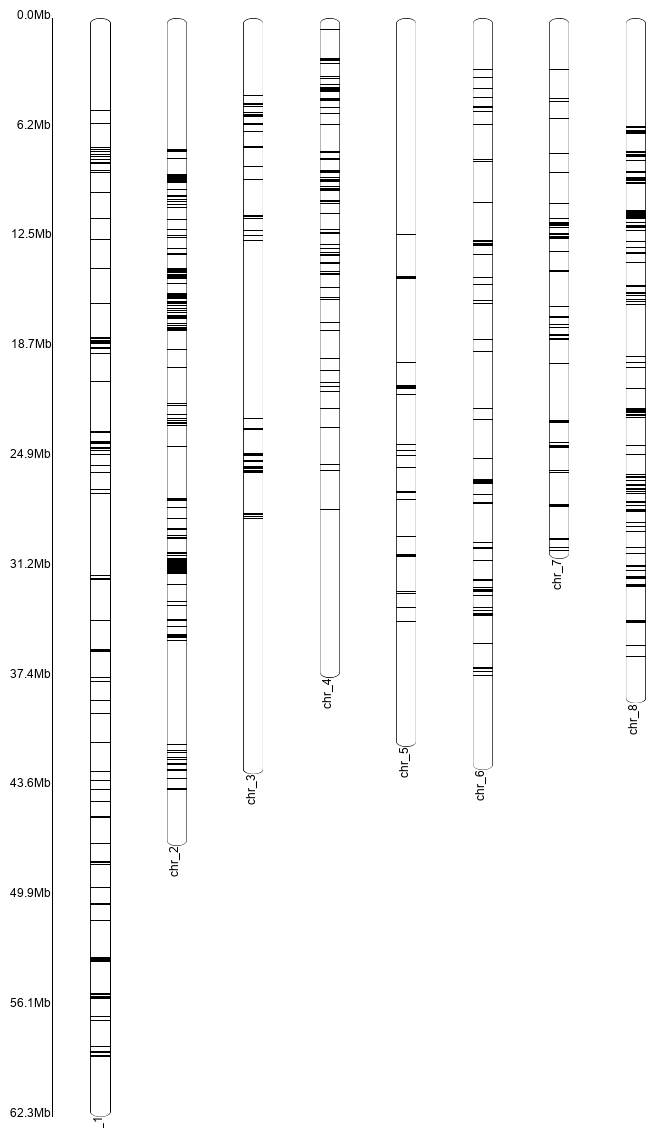


**Figure S6** Location of predicted *R* genes in the genome of sweet cherry cv. Tieton (*Prunus avium*).

**Table S1**. Statistics of sequencing data of Oxford Nanopore technology (ONT) and Illumina for sweet cherry (*Prunus avium*) cv. Tieton

| **Clean Reads** | **ONT Sequencing** | | **Illumina Sequencing** | |
| --- | --- | --- | --- | --- |
|  | **Raw reads** | **Clean reads** | **Raw reads** | **Clean reads** |
| **Size of library** | 20kb | 20kb | 350bp | 350bp |
| **Number of reads** | 5,399,643 | 4,073,577 | 321,826,964 | 316,873,360 |
| **Average length of reads** | 13,134 | 15,968 | 150 | 145.9 |
| **Total bases** | 70,922,674,823 | 65,048,910,929 | 48,274,044,600 | 46,240,402,940 |
| **MeanQuality** | 7.62 | 8.68 | - | - |
| **Sequencing depth** | 208x | 191x | 142x | 136x |

**Table S2**. Statistics of the sequencing and mapping results for sweet cherry cv. Tieton genome of Hi-C reads

| **Type** | | **Number** | |
| --- | --- | --- | --- |
| **Total Read Pairs** | | 134,298,049 | |
| **Total Bases** | | 40,192,529,496 | |
| **GC (%)** | | 40.72 | |
| **Q20 (%)** | | 97.06 | |
| **Q30 (%)** | | 92.38 | |
| **Mapped Reads** | | **Number** | **Percentage (%)** |
| **Total** | | 126,213,404 | 93.98 |
| **Unique Mapped Read Pairs** | **Valid Interaction Pairs** | 38,912,219 | 28.97 |
|  | **All intra Interaction Pairs** | 16,855,220 | 12.55 |
|  | **All inter Interaction Pairs** | 22,056,999 | 16.42 |
|  | **Duplicated Pairs** | 8,464,900 | 6.30 |
|  | **Total** | 47,377,119 | 35.28 |

**Table S3.** Long-terminal-repeat retrotransposon assembly index (LAI) analysis and Contig N50s of different genome assemblies in *Prunus*.

| **Genome** | **Contig N50 (kb)** | **Intact LTR-RT (%)** | **Total LTR-RT (%)** | **Raw LAI** | **LAI** |
| --- | --- | --- | --- | --- | --- |
| **Sweet cherry (*Prunus avium* cv. Tieton)** | 3.25 | 6.99 | 28.61 | 24.45 | 19.68 |
| **Sweet cherry (*Prunus avium* cv. Satonishiki)[**[**1**](#_ENREF_1)**]** | 0.22 | 0.35 | 11.60 | 2.98 | - |
| **Peach (*Prunus persica* v2.0)[**[**2**](#_ENREF_2)**]** | 0.25 | 3.78 | 20.04 | 18.86 | 18.79 |
| **Flower cherry (*Prunus yedoensis*)[**[**3**](#_ENREF_3)**]** | 0.13 | 2.04 | 20.31 | 10.05 | 7.08 |
| **Flower cherry (*Prunus Cerasus x yedoensis* v3.1)[**[**4**](#_ENREF_4)**]** | 0.92 | 2.97 | 23.73 | 12.50 | 6.87 |
| **Chinese plum (*Prunus mume*)[**[**5**](#_ENREF_5)**]** | 0.58 | 0.61 | 15.09 | 4.04 | 5.63 |
| **Almond (*Prunus dulcis* Lauranne v1.0)[**[**6**](#_ENREF_6)**]** | 0.082 | 2.34 | 19.00 | 12.32 | 11.86 |
| **Apricot (*Prunus armeniaca* )[**[**7**](#_ENREF_7)**]** | 1.02 | 2.63 | 18.02 | 14.57 | 16.29 |
| **European plum (*Prunus domestica*)[**[**8**](#_ENREF_8)**]** | 1.74 | 2.96 | 37.51 | 7.90 | 2.27 |

1. Shirasawa K, Isuzugawa K, Ikenaga M, Saito Y, Yamamoto T, Hirakawa H, et al. The genome sequence of sweet cherry (Prunus avium) for use in genomics-assisted breeding. DNA Res. 2017;24 5:499-508. doi:10.1093/dnares/dsx020.

2. Verde I, Jenkins J, Dondini L, Micali S, Pagliarani G, Vendramin E, et al. The Peach v2.0 release: high-resolution linkage mapping and deep resequencing improve chromosome-scale assembly and contiguity. BMC Genomics. 2017;18 1:225. doi:10.1186/s12864-017-3606-9.

3. Baek S, Choi K, Kim GB, Yu HJ, Cho A, Jang H, et al. Draft genome sequence of wild Prunus yedoensis reveals massive inter-specific hybridization between sympatric flowering cherries. Genome Biol. 2018;19 1:127. doi:10.1186/s13059-018-1497-y.

4. Shirasawa K, Esumi T, Hirakawa H, Tanaka H, Itai A, Ghelfi A, et al. Phased genome sequence of an interspecific hybrid flowering cherry, 'Somei-Yoshino' (Cerasus x yedoensis). DNA Res. 2019;26 5:379-89. doi:10.1093/dnares/dsz016.

5. Zhang Q, Chen W, Sun L, Zhao F, Huang B, Yang W, et al. The genome of Prunus mume. Nature communications. 2012;3:1318. doi:10.1038/ncomms2290.

6. Sanchez-Perez R, Pavan S, Mazzeo R, Moldovan C, Aiese Cigliano R, Del Cueto J, et al. Mutation of a bHLH transcription factor allowed almond domestication. Science. 2019;364 6445:1095-8. doi:10.1126/science.aav8197.

7. Jiang F, Zhang J, Wang S, Yang L, Luo Y, Gao S, et al. The apricot (Prunus armeniaca L.) genome elucidates Rosaceae evolution and beta-carotenoid synthesis. Horticulture research. 2019;6 1:128. doi:10.1038/s41438-019-0215-6.

8. Zhebentyayeva T, Shankar V, Scorza R, Callahan A, Ravelonandro M, Castro S, et al. Genetic characterization of worldwide Prunus domestica (plum) germplasm using sequence-based genotyping. Horticulture research. 2019;6:12. doi:10.1038/s41438-018-0090-6.

**Table S4**. Summary of the comparison between the annotation of genome of sweet cherry cv. Tieton and cv. Satonishiki.

|  | **cv. Tieton** | **cv. Satonishiki** |
| --- | --- | --- |
| **Total gene models** | 40,338 | 43,673 |
| **Total CDS length (bp)** | 43,836,807 | 39,418,633 |
| **Average CDS length (bp)** | 1086.74 | 902.59 |
| **Median CDS length (bp)** | 858 | 618 |
| **Annotation by GO** | 18,198 | 14,582 |
| **Annotation by KEGG** | 7,536 | 1,672 |

**Table S5**. Statistics of the repetitive sequences annotated in sweet cherry (*Prunus avium*) cv. Tieton genome

| **Type** | | **Number** | **Length (bp)** | **Percentage (%)** |
| --- | --- | --- | --- | --- |
| **DNA repeat elements** | Total | 23,321 | 12,980,715 | 3.77 |
|  | CMC-EnSpm | 6,887 | 6,304,192 | 1.80 |
|  | Crypton-S | 29 | 5,778 | 0 |
|  | Dada | 245 | 23,601 | 0.01 |
|  | MULE-MuDR | 4,227 | 2,488,633 | 0.72 |
|  | Maverick | 181 | 41,905 | 0.01 |
|  | Merlin | 80 | 20,890 | 0.01 |
|  | PIF-Harbinger | 2,516 | 1,164,858 | 0.34 |
|  | hAT | 215 | 28,248 | 0.01 |
|  | hAT-Ac | 3,470 | 1,131,742 | 0.33 |
|  | hAT-Tag1 | 2,090 | 759,211 | 0.22 |
|  | hAT-Tip100 | 2,239 | 773,141 | 0.22 |
|  | Unknown | 1,142 | 238,516 | 0.07 |
| **LINE** | Total | 7,363 | 3,262,996 | 0.94 |
|  | CR1 | 79 | 9,665 | 0.00 |
|  | L1 | 5,903 | 2,759,514 | 0.80 |
|  | L1-Tx1 | 1,251 | 478,135 | 0.14 |
|  | RTE-BovB | 130 | 15,682 | 0.00 |
| **LTR** | Total | 74,136 | 67,883,401 | 19.71 |
|  | Caulimovirus | 769 | 1,523,670 | 0.44 |
|  | Copia | 28,908 | 29,268,382 | 8.50 |
|  | ERV1 | 142 | 83,119 | 0.02 |
|  | ERVK | 1,591 | 285,301 | 0.08 |
|  | Gypsy | 19,450 | 26,140,435 | 7.59 |
|  | Ngaro | 144 | 56,139 | 0.02 |
|  | Pao | 780 | 962,402 | 0.28 |
|  | Unknown | 22,352 | 9,563,953 | 2.78 |
| Rolling-circle | Helitron | 2,094 | 1,175,738 | 0.34 |
| **SINE** | **Total** | **2,613** | **209,637** | **0.06** |
|  | B2 | 940 | 74,829 | 0.02 |
|  | ID | 1,673 | 134,808 | 0.04 |
| Unknown | | 212,076 | 114,542,377 | 33.27 |
| Low_complexity | | 14,899 | 760,396 | 0.22 |
| Satellite | | 465 | 118,368 | 0.03 |
| Simple_repeat | | 82,093 | 3,178,811 | 0.92 |
| RNA repeats | | 1,368 | 432,841 | 0.13 |
| Total | | 420,428 | 204,545,280 | 59.40 |

Note: LINE: Long interspersed nuclear elements, LTR: Long terminal repeat elements, SINE: Short interspersed nuclear elements

**Table S6**. Statistics of non-coding RNA prediction for sweet cherry (*Prunus avium*) cv. Tieton genome

| **Type** | **Number** | **Length (bp)** |
| --- | --- | --- |
| **tRNA** | 1,905 | 8,359 |
| **rRNA** | 621 | 272,467 |
| **miRNA** | 131 | 16,228 |
| **snRNA** | 1,114 | 124,159 |
| **Others** | 54 | 142,133 |

**Table S7**. Statistics of gene orthologs analysis between sweet cherry (*Prunus avium*) cv. Tieton and five other plant species in *Prunus*.

| **Type** | ***P.avium*** | ***P.yedoensis*** | ***P.persica*** | ***P.dulcis*** | ***P.mume*** | ***P.armeniaca*** | **Total** |
| --- | --- | --- | --- | --- | --- | --- | --- |
| Number of genes | 38,275 | 41,294 | 26,873 | 1,8169 | 31,390 | 30,436 | 186,437 |
| Number of genes in orthogroups | 36,385 | 35,904 | 25,109 | 1,7008 | 29,666 | 28,611 | 172,683 |
| Number of unassigned genes | 1,890 | 5,390 | 1,764 | 1,161 | 1,724 | 1,825 | 13,754 |
| Percentage of genes in orthogroups (%) | 95.1 | 86.9 | 93.4 | 93.6 | 94.5 | 94 | 92.6 |
| Percentage of unassigned genes (%) | 4.9 | 13.1 | 6.6 | 6.4 | 5.5 | 6 | 7.4 |
| Number of orthogroups containing species | 20,379 | 20,211 | 19,540 | 12,225 | 20,841 | 19,210 | 25,768 |
| Percentage of orthogroups containing species (%) | 79.1 | 78.4 | 75.8 | 47.4 | 80.9 | 74.5 | 100 |
| Number of species-specific orthogroups | 461 | 907 | 108 | 141 | 178 | 288 | 2,083 |
| Number of genes in species-specific orthogroups | 2,797 | 2,244 | 295 | 739 | 520 | 1,361 | 7,956 |
| Percentage of genes in species-specific orthogroups (%) | 7.3 | 5.4 | 1.1 | 4.1 | 1.7 | 4.5 | 4.3 |
| Number of orthogroups with all species present | 8,293 | | | | | | |
| Number of single-copy orthogroups | 3,282 | | | | | | |

Note: Only the longest transcript variant per gene was used for Orthofinder analysis.

**Table S8**. Statistics of gene family expansion and contraction

|  | Expansions | Genes Gained | Equal | Contractions | Genes Lost | Families Lost | Average Expansion | Significant Expansions | Significant Contractions |
| --- | --- | --- | --- | --- | --- | --- | --- | --- | --- |
| Pav | 1489 | 4070 | 8046 | 1909 | 2480 | 889 | 0.138937 | 108 | 13 |
| Par | 869 | 2557 | 7973 | 2602 | 3296 | 1334 | -0.0645753 | 75 | 14 |
| Pp | 1230 | 1828 | 7737 | 2477 | 3344 | 1455 | -0.132471 | 7 | 39 |
| Pyn | 5198 | 11184 | 4807 | 1439 | 2155 | 1023 | 0.788972 | 137 | 53 |
| Pm | 1472 | 2419 | 8921 | 1051 | 1344 | 432 | 0.0939357 | 44 | 8 |
| Pdu | 439 | 952 | 4309 | 6696 | 10167 | 4604 | -0.805225 | 9 | 11 |

Pav: *Prunus avium*, Pp: *Prunus persica*, Pd: *Prunus dulcis*, Par: *Prunus armeniaca*, Pm: *Prunus mume,* Pyn: *Prunus yedoensis.*

**Table S9**. KEGG pathways annotation of the significant expanded gene families in the genome of sweet cherry cv. Tieton.

| GeneID | KEGG | GeneID | KEGG | GeneID | KEGG |
| --- | --- | --- | --- | --- | --- |
| FUN_037397 | K17506 | FUN_003053 | K12869 | FUN_029333 | K08241 |
| FUN_002805 | K15014 | FUN_003056 | K12869 | FUN_029334 | K08241 |
| FUN_011831 | K14856 | FUN_003059 | K12869 | FUN_029335 | K08241 |
| FUN_014536 | K14856 | FUN_003060 | K12869 | FUN_029336 | K08241 |
| FUN_030680 | K14766 | FUN_005500 | K12869 | FUN_029337 | K08241 |
| FUN_008242 | K14508 | FUN_019923 | K12869 | FUN_009089 | K04079 |
| FUN_008243 | K14508 | FUN_019924 | K12869 | FUN_035868 | K04079 |
| FUN_008260 | K14508 | FUN_019925 | K12869 | FUN_009836 | K02872 |
| FUN_008591 | K14508 | FUN_019944 | K12869 | FUN_018011 | K02872 |
| FUN_019842 | K14508 | FUN_019945 | K12869 | FUN_018107 | K02872 |
| FUN_019843 | K14508 | FUN_025260 | K12869 | FUN_023676 | K02872 |
| FUN_019844 | K14508 | FUN_001099 | K10260 | FUN_028282 | K02872 |
| FUN_027349 | K14508 | FUN_000989 | K08248 | FUN_028468 | K02872 |
| FUN_039130 | K14508 | FUN_005329 | K08241 | FUN_030698 | K02872 |
| FUN_039137 | K14508 | FUN_005333 | K08241 | FUN_034499 | K02872 |
| FUN_039138 | K14508 | FUN_005335 | K08241 | FUN_034566 | K02872 |
| FUN_008261 | K14211 | FUN_029265 | K08241 | FUN_000107 | K02872 |
| FUN_038069 | K14211 | FUN_029266 | K08241 | FUN_004059 | K02872 |
| FUN_039088 | K14211 | FUN_029267 | K08241 | FUN_020392 | K02872 |
| FUN_028167 | K14196 | FUN_029269 | K08241 | FUN_024071 | K02872 |
| FUN_028957 | K13734 | FUN_029270 | K08241 | FUN_030349 | K02872 |
| FUN_017239 | K13264 | FUN_029273 | K08241 | FUN_034990 | K02872 |
| FUN_014730 | K13167 | FUN_029274 | K08241 | FUN_035899 | K02872 |
| FUN_009307 | K13116 | FUN_029275 | K08241 | FUN_036562 | K02872 |
| FUN_003041 | K12869 | FUN_029277 | K08241 | FUN_002437 | K01897 |
| FUN_019922 | K12869 | FUN_029278 | K08241 | FUN_035034 | K01674 |
| FUN_003029 | K12869 | FUN_029283 | K08241 | FUN_027960 | K05795 |
| FUN_003030 | K12869 | FUN_029284 | K08241 | FUN_035387 | K05795 |
| FUN_003037 | K12869 | FUN_029323 | K08241 | FUN_038231 | K04354 |
| FUN_003038 | K12869 | FUN_029324 | K08241 | FUN_003298 | K05795 |
| FUN_003043 | K12869 | FUN_029325 | K08241 | FUN_018122 | K05795 |
| FUN_003049 | K12869 | FUN_029327 | K08241 | FUN_020558 | K05795 |
| FUN_003050 | K12869 | FUN_029328 | K08241 | FUN_020650 | K05795 |
| FUN_003052 | K12869 | FUN_029332 | K08241 | FUN_026524 | K05795 |
| FUN_028290 | K15047  K15698 | FUN_036929 | K05641  K05643 | FUN_018625 | K02872  K03644 |
| FUN_023998 | K05641  K05643 | FUN_037199 | K05641  K05643 | FUN_020394 | K02872  K03644 |
| FUN_036835 | K05641  K05643 | FUN_037822 | K05641  K05643 | FUN_020478 | K02872  K03644 |
| FUN_036912 | K05641  K05643 | FUN_029017 | K04460  K15423 | FUN_028280 | K02872  K03644 |
| FUN_036928 | K05641  K05643 | FUN_003287 | K02872  K03644 | FUN_036698 | K02872  K03644 |
| FUN_010030 | K04558  K06867  K09255  K09257  K09259  K12271  K14508 | FUN_037314 | K04558  K06867  K09255  K09257  K09259  K12271  K14508 | FUN_017942 | K02872  K03644 |
| FUN_028378 | K00924  K08287  K08823 |  |  |  |  |

**Table S11**. Parameter settings for all genome assembly analysis tools

| **Tools** | **Parameter** |
| --- | --- |
| MaSuRCA | PE= pe 350 150 R1.fq.gz R2.fq.gz  <forward_reads> <reverse_reads>  NANOPORE=N01.filt.fa  CA_PARAMETERS = cgwErrorRate=0.15  FLYE_ASSEMBLY=1(For flye assembly, for CABO =0) |
| Canu | genomeSize=360m -nanopore-raw |
| wtdbg2 | -g 360m -x ont |
| NECAT | GENOME_SIZE=360000000, MIN_READ_LENGTH=3000, NUM_ITER=2, CNS_OUTPUT_COVERAGE=45 |
| BUSCO | -I embryophyta_odb10 |
| medaka | -m r941_prom_fast_g303 -b 80 |
| NextPolish | task = 121212, rerun = 3, sgs_options = -max_depth 100 –bwa |
| Purge Haplotigs | purge_haplotigs cov -l 58 -m 128 -h 217 |
| Arima-HiC Mapping | Default |
| ALLHiC | allhic extract --minLinks 50  allhic partition 8 --maxLinkDensity 3 --minREs 300 |
| Juicer | Default |
| 3D de novo assembly | Default |
| JupiterPlot | i=5 ng=95 m=200000 |
| LTRharvest | -minlenltr 100 -maxlenltr 7000 -mintsd 4 -maxtsd 6 -motif TGCA -motifmis 1 -similar 85 -vic 10 -seed 20 -seqids yes |
| LTR_retriever | Default |
| Jellyfish | -m 37 |
| GenomeScope | genomescope.R 37 150 |
| RepeatModeler | -LTRStruct |
| RepeatMasker | -e ncbi -lib RepeatModeler_classified |
| Funannotate | mask -m repeatmasker --repeatmasker_species Embryophyta  train --left 1.fastq --right 2.fastq --stranded RF --max_intronlen 50000  predict --busco_seed_species arabidopsis --busco_db embryophyta --max_intronlen 50000 --organism other --repeats2evm -w augustus:6  annotate --busco_db embryophyta |
| InterProScan5 | Default |
| EggNOG-mapper | -m diamond |
| KAAS | BLAST for Eukaryotes BBH |
| Infernal | Default |
| tRNAscan-SE | Default |
| RNAmmer | -S euk -m lsu,ssu,tsu |
| OrthoFinder | Default |
| CAFÉ | tree (Pdu:68.2,(Pp:33.2564,((Pyn:18.2068,Pav:18.2068):7.96885,(Pm:17.9145,Par:17.9145):8.26111):7.08074):34.9436) |
| python version of MCscan | jcvi.compara.synteny screen --minspan=5 |

Note: We only listed the changed parameters in the table, otherwise use the default parameters.

**Table S12**. Statistics of protein-coding gene prediction for sweet cherry (*Prunus avium*) cv. Tieton genome

| **Prediction Pipeline** | **Number of genes** | **Weight for EVidenceModeler** |
| --- | --- | --- |
| **Augustus** | 26,750 | 6 |
| **Augustus HiQ** | 12,295 | 2 |
| **CodingQuarry** | 78,577 | 2 |
| **GeneMark** | 186,938 | 1 |
| **GlimmerHMM** | 84,836 | 1 |
| **PASA** | 18,876 | 6 |
| **SNAP** | 65,310 | 1 |
| **Total** | 473,582 | - |

PASA: Program to Assemble Spliced Alignments. Augustus HiQ: high quality predictions from Augustus
